# Basic Leucine Zipper Transcription Factors as Important Regulators of Leydig Cells’ Functions

**DOI:** 10.3390/ijms232112887

**Published:** 2022-10-25

**Authors:** Luc J. Martin, Ha Tuyen Nguyen

**Affiliations:** Biology Department, Université de Moncton, Moncton, NB E1A 3E9, Canada

**Keywords:** activator protein 1, CREB, CEBPB, testis, Leydig cells, steroidogenesis

## Abstract

Transcription factors members of the basic leucine zipper (bZIP) class play important roles in the regulation of genes and functions in testicular Leydig cells. Many of these factors, such as cAMP responsive element binding protein 1 (CREB1) and CCAAT enhancer binding protein beta (CEBPB), are regulated by the cAMP/protein kinase A (PKA) pathway, the main signaling pathway activated following the activation of the luteinizing hormone/choriogonadotropin membrane receptor LHCGR by the - hormone LH. Others, such as X-box binding protein 1 (XBP1) and members of the cAMP responsive element binding protein 3 (CREB3)-like superfamily, are implicated in the endoplasmic reticulum stress by regulating the unfolded protein response. In this review, the influences of bZIP transcription factors, including CREB1, CEBPB and activator protein 1 (AP-1) family members, on the regulation of genes important for cell proliferation, steroidogenesis and Leydig cell communication will be covered. In addition, unresolved questions regarding the mechanisms of actions of bZIP members in gene regulation will be identified.

## 1. Introduction

There is increasing evidence that transcription factors of the basic leucine zipper (bZIP) class play important roles in gene regulation and function of testicular Leydig cells. This class of more than 50 members includes the cAMP responsive element binding protein/activating transcription factor (CREB/ATF), activator protein 1 (AP-1), CCAAT enhancer binding protein (CEBP) and musculoaponeurotic fibrosarcoma (MAF) families of transcription factors. The bZIP transcription factors are proteins found only in eukaryotes. They bind to specific double-stranded DNA sequences as homodimers or heterodimers to activate or repress gene transcription. Most of the genes encoding bZIP factors are classified as immediate early genes and are inducible by a multitude of endocrine and paracrine agents leading to the activation of intracellular signaling pathways. The primary stimulatory hormone acting on Leydig cells is luteinizing hormone (LH) from the anterior pituitary gland. This hormone interacts with its membrane receptor luteinizing hormone/choriogonadotropin (LHCGR) and mostly activates the cAMP/protein kinase A (PKA) pathway. Then, PKA phosphorylates a multitude of transcription factors, including bZIP members, leading to their activation and regulation of transcription of their target genes. Although the roles of certain bZIP transcription factors, such as CREB1, CEBPB, JUN and FBJ osteosarcoma (FOS), are well documented regarding the regulation of steroidogenesis in Leydig cells, other lesser-known members may play complementary roles in the regulation of proliferation, survival, and communication in these cells. Thus, this review aims to present recent advances regarding the involvement of bZIP factors in the regulation of different functions of Leydig cells from the testis.

## 2. Classification of bZIP Transcription Factors

**Figure 1:**
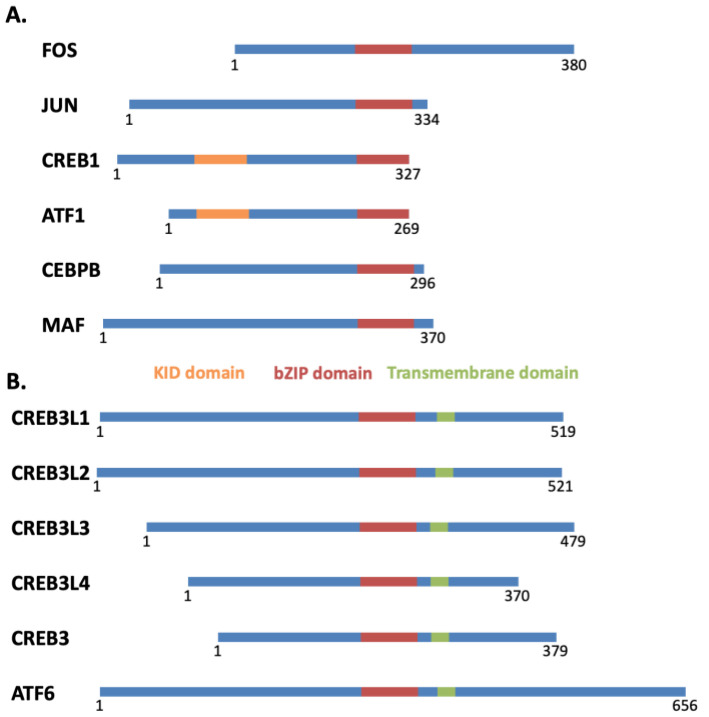
Schematic diagram of the primary protein structure of transcription factors members of the bZIP class. (**A**) Common nuclear bZIP transcription factors. (**B**) Transmembrane bZIP transcription factors. Abbreviations: KID, kinase A inducible activation domain; bZIP, basic leucine zipper.

Transcription factors members of the bZIP class are characterized by the presence of a bZIP domain consisting of an approximately 40 amino acid-long α-helix (Figure 1). The N-terminal third of this helix has multiple positively charged side chains such as arginine and lysine and is defined as the basic region involved in DNA-binding. The C-terminal two-thirds of the helix is the leucine zipper and plays a role in protein dimerization. The leucine zipper is amphipathic with the hydrophobic regions on the two copies of the leucine zipper interacting to bring the two bZIP domains together in a parallel orientation. The two basic regions can then bind to DNA by passing through the major groove on opposite sides of the double helix. Importantly, the bZIP domain facilitates the dimerization of two similar transcription factors of the bZIP class. The combination of two transcription factors containing the bZIP domain enables the proper positioning of the two adjacent DNA-binding domains in the dimeric complex. For bZIP transcription factors, dimerization is required for DNA binding.

According to the latest TFClass nomenclature, the class of bZIP transcription factors contains more than 50 members divided into families: JUN, FOS, MAF, B-ATF, X-box binding protein 1 (XBP1), ATF4, CREB and CEBP (Table 1) [1]. The common AP-1 family of transcription factors rather consists of members of the JUN and FOS subfamilies, whereas the common CREB/ATF family refers to CREB1, ATF1 and cAMP responsive element modulator (CREM) transcription factors.

## 3. Mechanisms of Gene Regulation by bZIP Transcription Factors

### 3.1. CREB-like Subfamily Members

**Figure 2:**
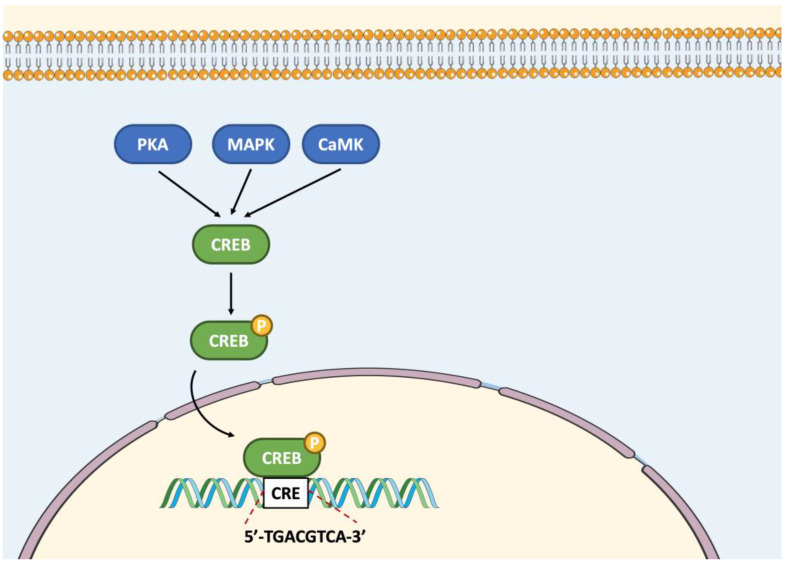
Regulation of the activity of CREB-like subfamily members by phosphorylation, followed by recruitment to the CRE DNA regulatory element. Abbreviations: CaMK, Ca^2+^/calmodulin dependent protein kinase; CRE, cyclic AMP response element; CREB, cAMP responsive element binding protein; MAPK, mitogen activated protein kinase; P, phosphate; PKA, protein kinase A.

CREB1 is a well-known 43 kD bZIP transcription factor that binds to an octanucleotide sequence called the cyclic AMP response element (CRE) (5′-TGACGTCA-3′) (Figure 2) [2]. This factor can bind to DNA as a homodimer or a heterodimer in association with other members of the CREB/ATF and AP-1 families [3]. CREB1 plays an essential role in the regulation of gene expression in response to a variety of extracellular signals. In response to a cytosolic increase in the second messenger cAMP, CREB can be phosphorylated by PKA at serine residue Ser^133^ in the kinase A inducible activation domain (KID) (Figure 2) [2]. This phosphorylation enhances CREB1 transcriptional activity by facilitating its interaction with the 265 kD CREB-binding protein (CBP) and subsequent recruitment of CBP to target genes [4]. This cofactor is capable of histone acetyltransferase activity and acts as an adaptor protein between CREB and the general transcription complex.

The CREB-like subfamily members CREB1, CREM and ATF1, are expressed in the MA-10 tumor Leydig cell model [5]. In testicular Leydig cells, the constitutively expressed transcription factor CREB1 represents one of the most important targets of the cAMP/PKA pathway. Among the target genes of CREB1 in Leydig cells is *Nr4a1* encoding the orphan nuclear receptor NR4A1 (NUR77). Indeed, the rapid activation of *Nr4a1* gene expression in response to LH in Leydig cells is caused, in part, by CREB1 and AP-1 transcription factors following activation of the cAMP/PKA pathway [6]. In addition to being activated by PKA, CREB1 can also be activated by the Ca^2+^/calmodulin-dependent protein kinase (CaMK), the mitogen-activated protein kinase (MAPK) and/or AKT through phosphorylation at Ser^133^ in other cell types (Figure 2) [7,8,9,10]. By binding to a G protein-coupled receptor, the osteoblast-derived hormone osteocalcin also regulates steroidogenesis in Leydig cells following activation of CREB1 [11].

Although the importance of protein kinase C (PKC) is less than that of PKA, these two pathways appear to potentiate each other in the activation of steroidogenesis in response to LH in Leydig cells [12,13,14]. In addition to contributing to its activation, phosphorylation of CREB1 by PKC isoenzymes such as PKCµ (PKD) increases its ability to bind to CRE regulatory element [2,15]. Moreover, PMA (phorbol-12-myristate-13-acetate), an activator of the PKC pathway, increases the phosphorylation of CREB1 [12,16].

In addition to CREB1, cAMP/PKA-mediated increase in transcription can also involve activation of CREM (cAMP-response element modulator protein) by phosphorylation at Ser^117^ and its binding to CBP [17]. Alternative splicing of the *Crem* transcript can give rise to several isoforms which act as activators (τ, τ1, and τ2) or repressors (α, β, and γ) of transcription [18]. Among activators, CREMτ can interact with the steroidogenic factor 1 (NR5A1) and activate the steroidogenic acute regulatory protein (*Star*) promoter following treatment of MA-10 Leydig cells with dibutyryl-cAMP [19]. In addition, transcriptional activity of CREM, like that of CREB1, is increased post-translationally by phosphorylation following activation of PKA in response to cAMP [17]. The *Crem* gene contains a promoter that can be activated by the cAMP/PKA pathway. Such an activation results in the production of transcripts encoding a particular isoform of CREM which contains the DNA binding domain and the phosphorylatable region, but lacks the activation domain [20]. This type of isoform, called ICER (inducible cyclic AMP early repressor), binds to the CRE regulatory element and repress transcriptional activation by the active isoform of CREM, thereby limiting cAMP/PKA-dependent activation of target genes [21].

ATF1, another member of the CREB-like subfamily, can dimerize with CREB1 but not with ATF2 or ATF3, whereas ATF2 can dimerize with ATF3 but not with CREB1 [22,23,24]. In addition, certain CRE-binding proteins, including ATF2, ATF3 and ATF4, can heterodimerize with FOS and JUN [24,25]. Interestingly, ATF1-4 are all expressed in adult human Leydig cells [26] and may participate in the regulation of steroidogenesis. ATF1 can be phosphorylated at Ser^63^ by PKA or CAMK [27], potentially contributing to its transcriptional activity in Leydig cells.

### 3.2. AP-1 Members

**Figure 3:**
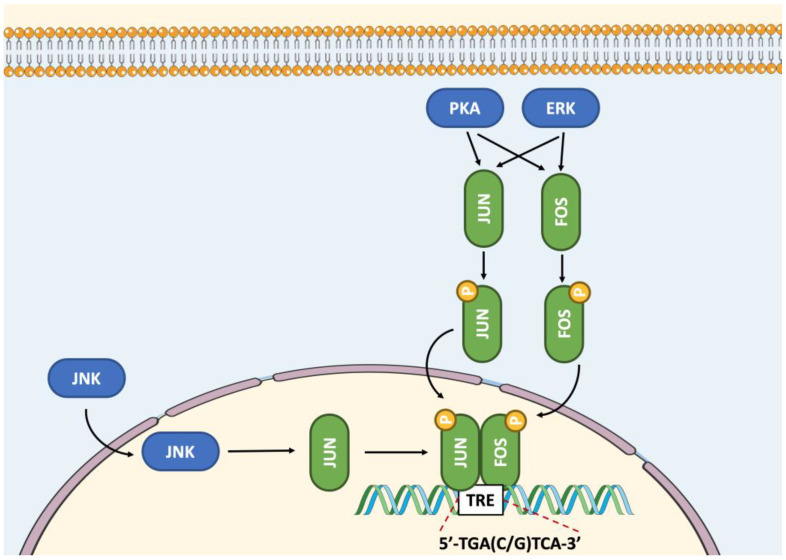
Regulation of the activity of AP-1 family members JUN and FOS by phosphorylation, followed by recruitment to the TRE DNA regulatory element. Abbreviations: ERK, extracellular signal-regulated kinase; JNK, JUN N-terminal kinase; P, phosphate; PKA, protein kinase A; TRE, 12-O-tetradecanoylphorbol-13-acetate response element.

The AP-1 family of transcription factors, including members of the JUN and FOS subfamilies, is ubiquitously expressed [28]. FOS subfamily members, including FOS, FOSB, fos-like antigen 1 (FOSL1, FRA1) and fos-like antigen 2 (FOSL2, FRA2) must heterodimerize with JUN proteins, including JUN, JUNB and JUND. However, JUN members can form homodimers or heterodimers with other AP-1 factors. Overall, JUN-FOS heterodimers bind DNA with more affinity and stability than JUN-JUN homodimers [29,30]. AP-1 members were involved in the induction of multiple cellular and viral genes through the phorbol ester tumor promoter 12-O-tetradecanoylphorbol-13-acetate response element (TRE) (Figure 3) [31]. With other bZIP transcription factors such as ATF and MAF, AP-1 members can form up to 18 different DNA-binding dimeric complexes with versatile functionality through activation or inhibition of transcription according to the composition of the dimeric complex and the cell context [32,33]. Despite having comparable DNA binding specificities, AP-1 dimers differ in their transactivation efficiencies [34,35]. Unlike FOS and FOSB, FOSL1 and FOSL2 lack transcriptional activation domains.

Regarding their interaction with DNA regulatory elements, JUN:FOS and JUN:JUN dimers can bind to AP-1 or TRE elements with the consensus sequence 5′-TGA(C/G)TCA-3′ and to CRE [28]. Notably, JUN:FOS heterodimers have higher affinity for asymmetrical TRE motifs, whereas JUN homodimers rather bind to symmetric CRE elements [29,36]. The heterodimers formed from ATF members typically bind to CRE [3].

There may be functional redundancy among AP-1 members. Indeed, FOS, FOSB, and JUND are dispensable but JUN, JUNB, and FOSL1 are necessary for embryonic development [37]. Furthermore, substitution of *Jun* for *Junb* allows recovery of liver development but not heart development, suggesting that knock-in experiments are not equivalent for all AP-1 members [38].

There are several ways to activate and/or increase the expression of AP-1 factors. In fact, numerous signaling pathways leading to the phosphorylation of serine and threonine residues participate in the regulation of their expression and activity [39,40]. The *Jun* promoter is primarily activated by the recruitment of JUN/ATF2 heterodimer to a major Jun/TRE *cis* regulatory element [41]. Three regulatory elements—the sis-inducible enhancer (SIE), the serum response element (SRE), and the CRE—can activate the *Fos* promoter in response to a variety of hormones, growth factors, and cytokines [33,42,43,44,45,46]. Indeed, the expression of *Fos* will be enhanced following the recruitment of ATF or CREB factors to a CRE element in response to increased levels of second messengers cAMP and calcium [8,44]. The SRE regulatory element can be bound by a dimer consisting of the serum response factor (SRF) and ETS Like-1 (ELK1) being activated by exposure to UV light or growth factors, leading to increased *Fos* expression [46,47]. The SIE regulatory element allows the recruitment of dimers of the signal transducer and activator of transcription (STAT) family, in particular STAT1 and STAT3, to activate *Fos* gene expression [45,48]. These STAT members are mainly activated by the Janus kinase (JAK) pathway [49]. In most cell types, the SRE and CRE in the *Fos* and AP-1 (TRE) element in the *Jun* promoters are involved in basal expression of these genes [50,51,52]. Moreover, *Jun* is autoregulated by its own expression with the JUN/ATF2 heterodimer [41,51]. Similarly, the *Fos* gene can also self-regulate after its activation [53]. Hence, AP-1 members can regulate each other to fine-tune their expression [54].

The MAPK pathway primarily regulates the activity of AP-1 members, through phosphorylation by extracellular signal-regulated kinases (ERK), JUN N-terminal kinases (JNK), and p38 (Figure 3). In addition, other kinases such as glycogen synthase kinase-3 (GSK3), p34^cdc2^ kinase (CDK1), casein kinase II (CKII), PKA and PKC can phosphorylate FOS or JUN in certain cell types. The activated JNK translocates to the nucleus [47], where it phosphorylates AP-1 members, promoting their dimerization and transcriptional activities [33]. Interestingly, the dimerization and DNA binding of AP-1 members facilitate their phosphorylation by several protein kinases [40]. Different kinases participate in the regulation of the activity of AP-1 members by phosphorylating different Ser or Thr residues. For example, the phosphorylation of JUN at Thr^235^, Ser^246^ and Ser^252^ by GSK3, CKII or ERK1/2 decreases its transcriptional activity [39,55,56,57]. However, phosphorylation at Ser^63^ and Ser^73^ by JNK1 and dephosphorylation at Ser^246^ increase the stability and transcriptional activity of the JUN protein [39,55]. Such post-translational modifications of JUN potentiate its transactivation capacity by increasing its interaction with the coactivator CBP, having histone acetyltransferase activity [58]. Similar to JUN, JUNB can also be phosphorylated at Thr^102/104^ by ERK2 [59,60]. However, the consequence of this post-translational modification remains to be defined. The phosphorylation of FOS at Ser^362^, Ser^374^, Thr^232^, Thr^325^, and Thr^331^ by ERK and ribosomal S6 kinase increases its protein stability and transcriptional activity [61,62,63]. Moreover, FOS can also be phosphorylated at Ser^362^ by PKA [64]. Compared to other AP-1 factors, JUND cannot interact directly with JNK, but can be phosphorylated by this kinase as part of a heterodimer with JUN [65]. Hence, in addition to changes in FOS and JUN protein levels, gene regulation can be influenced by their post-translational modifications (Figure 3).

In addition to LH and human chorionic gonadotrophin (hCG), several growth factors are also involved in the activation of AP-1 family members in Leydig cells. In Leydig cells from 3-week-old pigs, basic fibroblast growth factor (bFGF) and epidermal growth factor (EGF) increase the expressions of *Fos*, *Jun*, and *Junb*, whereas transforming growth factor beta (TGF) increases the expression of *Jun* only, and insulin-like growth factor 1 (IGF1) increases the expressions of *Fos* and *Junb* [66]. Interestingly, EGF and bFGF enhance the stimulatory action of hCG on *Fos* and *Junb* expressions in this model [66]. Regulation of AP-1 members in Leydig cells can also be influenced by testicular macrophages and tumor necrosis factor alpha (TNFα) production through regulation of members of the MAPK family such as ERK [67] and stress-activated protein kinases (SAPKs) [68]. In MA-10 Leydig cells, the SAPK protein level and activity are increased, whereas those of ERK are decreased, by TNFα and cAMP [69]. Such a regulation results in increases in JUN and FOS protein levels and DNA binding activities [69]. In addition, others have shown that CREB nuclear localisation is decreased in response to TNF, resulting in inhibition of steroidogenesis in MA-10 Leydig cells [70].

Although JUN and FOS are expressed in MA-10 Leydig cells, only JUN is rapidly increased in response to activation of the cAMP/PKA pathway [71,72]. However, hCG increases the expressions of *Fos*, *Fosb*, *Jun*, *Junb*, *Jund* and *Fosl2* in less than 1 h of treatment, whereas *Fosl1* is increased after 3 h of exposure of MA-10 Leydig cells [73]. In addition to the cAMP/PKA pathway, ERK1/2 and PKC are also involved in the activation of AP-1 members by LH/hCG stimulation of testicular Leydig cells [15,74,75].

### 3.3. CEBP Members

The CEBP subfamily includes six members: CEBPA, CEBPB, CEBPG, CEBPD, CEBPE and DNA-damage inducible transcript 3 (DDIT3, CHOP) [76]. CEBPB is the major member expressed in Leydig cells and its expression depends on Leydig cells’ differentiation status [77]. Members of the CEBP subfamily can form homodimers or heterodimers with members of the CREB/ATF family via the leucine zipper dimerization domain [78], which greatly increases the diversity of DNA binding specificities and transactivation potential. CEBP members can recognize the consensus DNA-binding sequence 5′-TKNNGYAAK-3′ (Y = C or T, K = T or G) in the regulatory regions of target genes. CEBPB, whose expression is increased in response to LH and cAMP, plays an important role in hormonal regulation of gene expression in Leydig cells [77]. In human, DNA-binding of CEBPB is increased by ERK-dependent phosphorylation of Thr^235^ [79]. In addition, Ca^2+^-mediated signals can also lead to mouse CEBPB activation through phosphorylation of Ser^276^ in the leucine zipper [74]. Acetylation and sumoylation have also been implicated in the regulation of intracellular localization and transcriptional activity of CEBP members [75,80] but have been barely investigated in the regulation of Leydig cells’ functions.

### 3.4. Maf-Related Members

The family of MAF-related transcription factors can be divided in two sub-families: the large MAF, containing MAF, MAFA, MAFB and neural retina leucine zipper (NRL), and the small MAF, containing MAFF, MAFG and MAFK (Table 1). The heterodimers formed by MAF factors bind to MAF recognition elements (MAREs) consisting of extended sequences of the AP-1 motifs [81]. Depending on the binding site and binding partner, MAF-related members can act as transcriptional activators or repressors. By E14.5 in mouse embryo and in adult testis, *Mafb* expression is detected in Leydig cells [82,83]. However, the target genes of MAFB in fetal Leydig cells during development or in adult Leydig cells remain to be characterized. Other MAF-related members may play an important role in regulating adult Leydig cells’ functions as MAF, MAFF and MAFG are highly expressed in human adult Leydig cells [26].

### 3.5. XBP1-Related Members

The XBP1-related members family contains only the transcription factor XBP1. This transcription factor is known to regulate the major histocompatibility complex (MHC) class II gene by binding to a promoter element known as X-Box [84]. XBP1 is implicated in cell differentiation, proliferation, apoptosis, cellular stress response and other signaling pathways. It has also been associated with the expression of genes required for membrane biogenesis and the secretory pathway [85]. Indeed, XBP1-deficient β-cells fail to secrete enough insulin to regulate blood glucose levels [86]. Several publications have evaluated ATF4 and XBP1 transcription factors as endoplasmic reticulum (ER) stress markers in Leydig cells [87,88]. With ATF4 and ATF6, XBP1 has been characterized as an unfolded protein response (UPR) transcription factor, which mediates downregulation of the 3-β-hydroxysteroid dehydrogenase (*Hsd3b1*) gene expression following high or prolonged hCG treatment in Leydig cells [89]. Such treatments result in ER stress-mediated apoptosis, possibly through binding to the ER stress response element (ERSE) found in different gene promoters [90]. Additionally, XBP1 can also form heterodimers by interacting with FOS [91] and ATF6 [92]. However, other specific target genes for XBP1 in Leydig cells remain to be further characterized.

### 3.6. CREB3-like Subfamily Members

The CREB3-like subfamily includes transmembrane bZIP transcription factors such as CREB3 (also known as Luman), CREB3L1, CREB3L2, CREB3L3 and CREB3L4. These transcription factors play important roles in the UPR. In addition, CREB3L1 is involved in differentiation, function, and survival of many cell types in which it is expressed. CREB3L1 can interact with other members of this subfamily such as itself and CREB3L3, as well as with CREM [78]. Targets for CREB3L1 include genes crucial for ER stress such as XBP1 and the chaperone heat shock protein 5 [93]. In addition to forming a heterodimer with CREB3L1, CREB3L3 can also heterodimerize with ATF6 and CEBPG [78,94]. Interestingly, CREB3L2 and CREB3L3 can be phosphorylated by cyclin-dependent kinases and influence cell division [63,95,96].

### 3.7. ATF-4 Related Members

ATF family members play essential roles in cell proliferation and differentiation, apoptosis, and inflammation. All members of this family, including ATF1, ATF2, ATF3, ATF4, ATF5, ATF6 and ATF7, can be detected in human Leydig cells [26]. However, their roles and functions in the regulation of gene expression in these cells remain to be determined. Like CREB1, ATF1 is involved in the regulation of transcription in response to variations in intracellular cAMP [97]. ATF2 and ATF3 regulate the expression of stress-response genes [98]. Interestingly, these ATF members can heterodimerize with transcription factors of the AP-1 family [3,24,99,100] to regulate gene expression. ATF4 is induced by stress signals including hypoxia, oxidative stress, ER stress and amino acid deprivation [101]. This transcription factor can bind to asymmetric CRE elements as a heterodimer and palindromic CRE elements as a homodimer. With DDIT3, ATF4 activates the transcription of the integrated stress response regulator tribbles pseudokinase 3 (TRIB3) and promotes ER stress-induced apoptosis of neuronal cells [102]. Interestingly, TRIB3 is highly expressed in different Leydig cell lines such as MA-10 and LC540 [103,104]. TRIB3 expression is rapidly modulated by xenobiotics, suggesting that its transcription may be regulated by immediate-early transcription factors like ATF4. Of interest for Leydig cells, ATF4 can heterodimerize with CEBPB [95] or JUN [3]. However, their target genes remain to be characterized. In addition, the activity of ATF4 can be modulated by phosphorylation at Ser^69^ [96,105], however the kinase involved and its functionality in Leydig cells remain to be determined.

ATF5 can bind to a ATF5-specific response element (ARE) (consensus: 5′-CYTCTYCCTTW-3′) or an amino acid response element (AARE) (consensus: 5′-WTTGCATCA-3′) present in many promoters. This transcription factor is involved in the regulation of cell survival, proliferation and differentiation [99,106]. Precisely, its transcriptional activity is enhanced by cyclin D3 and is inhibited by cyclin-dependent kinase 4 [106], suggesting that ATF5 may be an important regulator of Leydig cells’ proliferation.

Similar to CREB3-like subfamily members, ATF6 is involved in the regulation of genes important for ER stress response [100]. These transcription factors contain a transmembrane domain, allowing their localization to the ER in the absence of activation of the UPR.

In general, members of the bZIP class of transcription factors are involved in tumorigenesis and normal physiological processes like cell proliferation, development, steroid synthesis, and cell-to-cell interactions.

## 4. bZIP Transcription Factors and Leydig Cell Proliferation and Development

Before reaching the adult stage, progenitor Leydig cells must proliferate and differentiate in the testicular interstitium to acquire their full capacity for androgen synthesis. Stem Leydig cells (SLC) proliferate and differentiate into progenitor Leydig cells (PLC) between postnatal day (P)7 and P14 in the rat. PLC are the major Leydig cells of the testis from P14 to P21 [107]. At around P35, the immature Leydig cells (ILC) population is established from the differentiation of PLC and mainly produces androstanediol (3α-Diol) as androgen. By the end of puberty (from P55 to P90), adult Leydig cells (ALC) originate from the proliferation and differentiation of ILC [108], and are characterized by high levels of LHCGR and expression of the enzyme HSD3B1 [109]. These changes contribute to the increased testosterone synthesis from ALC [110] and are essential for normal reproductive function [111] and spermatogenesis [112]. Once differentiated, ALCs are primarily involved in two processes important to support spermatogenesis: testosterone production, and INSL3 synthesis and secretion.

### 4.1. CREB-Related Members

Among CREB-related transcription factors, ATF1 may promote gene expression, contributing to Leydig cells’ proliferation. Indeed, cyclin-dependent kinase 3 has been reported to phosphorylate ATF1 at Ser^63^, enhancing transactivation capacity of ATF1 and promoting cell proliferation [113]. CREB3 is a transcriptional coregulator involved in ER-stress. In mouse Leydig tumor cells (MLTC-1), the shRNA-mediated knockdown of CREB3 decreased the percentage of S phase cells and increased the expressions of cyclin A1, B1 and D2, leading to reduced apoptosis and increased cell proliferation [114]. However, the use of tumor Leydig cell lines to study normal Leydig cell proliferation may not be appropriate, and the interpretation of the data should be done with caution.

### 4.2. AP-1 Members

AP-1 members are well known for their ability to regulate cell proliferation. Indeed, JUN and FOS regulate the expression of cyclin D1, an important activator of cyclin-dependent kinases that promote cell cycle progression [115]. JUNB and JUND, in contrast to JUN, negatively regulate cell proliferation. In fact, JUNB inhibits JUN-mediated cyclin D1 expression [116], whereas JUND activation results in decreased fibroblasts’ proliferation [117]. The p21-activated protein kinase (PAK)2-dependent phosphorylation of JUN also contributes to EGF-mediated cell proliferation and transformation [118]. Since AP-1 members can regulate the expression of genes important for cell proliferation and differentiation [37,119,120,121], these transcription factors may influence Leydig cells’ development and maturation. Interestingly, *Jun* and *Fos* are highly expressed in PLC, and like *Junb*, *Jund* and *Fosl2* show a decreased expression upon maturation of PLC to ALC [122]. In addition, *Jun* and *Fos* are highly expressed in rat mesenchymal cells involved in the regeneration of ALC [123].

Being highly expressed in PLC and ILC [124], the androgen receptor (AR) is critical for proliferation and differentiation of these cells into adult Leydig cells. Indeed, testicular feminized (*Tfm*) male mice, having a non-functional mutated androgen receptor, contain Leydig cells with reduced levels of LHCGR, are non-responsive to hCG and have decreased testosterone production due to reduced cytochrome P450 family 17 subfamily A member 1 (CYP17A1) levels [110,125]. In addition, mice with Leydig cell-specific inactivation of *Ar* by *Cre* expression under the control of the *Amhr2* promoter [126,127] produced less testosterone and were infertile due to disrupted spermatogenesis [128]. Proliferation and differentiation of PLC and ILC may require AP-1 members, since JUN can interact with AR [129,130] and can influence its transcriptional activity. However, such modulation of AR activity by AP-1 members remains to be confirmed in Leydig cells.

As suggested previously, increased ATF2 activity under pathophysiological conditions such as diabetes may increase caspase-3 activity and promote Leydig cells’ apoptosis, leading to male hypogonadism [131]. ATF2, a transcription factor downstream of p38 MAPK, is a key mediator of extracellular stimulus-induced testicular injury. In addition, ATF2 can interact with AR [132,133], potentially modulating its transcriptional activity and influencing Leydig cells’ proliferation.

### 4.3. CEBP-Related Members

During differentiation of SLC into ALC, the expression of *Alkbh5*, coding for an RNA demethylase, is increased, leading to reduced levels of N^6^-methyladenosine methylation and increased autophagy in Leydig cells [134]. Interestingly, *Alkbh5* expression is increased in response to hCG by recruitment of CEBPB to its promoter region [134]. In SLCs, CEBPB also regulates the expression of *Ptgs2*, encoding the cyclooxygenase 2 enzyme which can modulate cell proliferation and apoptosis [135]. Thus, this bZIP transcription factor indirectly contributes to Leydig cell maturation by influencing mRNA methylation levels and the production of prostaglandins. DDIT3 can act as a dominant-negative inhibitor by forming a heterodimer with other CEBP members and preventing their DNA binding activity [136]. DDIT3 is activated by ER stress and promotes apoptosis [102,137]. Interestingly, decreased expression of DDIT3 is required for CEBPB to upregulate *Ptgs2* expression during SLC maturation [136].

As with other proline and acidic amino acid-rich (PAR) subfamily members, the bZIP transcription factor D site albumin promoter binding protein (DBP) is involved in the regulation of cell circadian rhythm, as reported with β-cells and the production of insulin [138]. Interestingly, the expression of DBP is inhibited by the mycotoxin zearelenone in Leydig cells [139]. However, further investigation will be required to better define its implication in regulation of proliferation and steroidogenic genes in Leydig cells.

### 4.4. Transmembrane bZIP Transcription Factors

In response to ER stress, cells initiate the UPR to restore protein homeostasis. The UPR is distinguished by three signaling proteins named protein kinase R (PKR)-like endoplasmic reticulum kinase (PERK), ATF6, and inositol-requiring enzyme 1 alpha (IRE1α). Activation of PERK leads to phosphorylation of eukaryotic initiation factor 2α, and then induces the bZIP transcription factor ATF4, causing increased expression of the pro-apoptotic bZIP protein DDIT3 and cell death [140]. On the other hand, the bZIP transcription factor ATF6 transits to the Golgi where it is cleaved into an activated form [140]. In addition, activated IRE1 catalyzes the splicing of a small intron from XBP1 mRNA to produce an active transcription factor. ATF6 and IRE1 pathways may also induce DDIT3 expression to trigger apoptosis [141]. Thus, ER stress can activate different bZIP transcription factors, leading Leydig cells to undergo apoptosis [89].

Overall, several transcription factors of the bZIP class influence the proliferation, maturation, and apoptosis of adult Leydig cells. However, their involvement in the differentiation of fetal Leydig cells remains to be demonstrated.

## 5. bZIP Transcription Factors and Steroidogenesis

Regulation of steroidogenesis in ALC is primarily mediated by the hypothalamic-pituitary-gonadal axis through LH secretion and binding to its LHCGR, resulting in de novo protein synthesis [142]. Following activation of its receptor, LH mainly stimulates cAMP production by adenylate cyclase, which leads to activation of PKA enzyme and upregulation of several genes contributing to increased androgen synthesis by Leydig cells. Members of the bZIP class being phosphorylated by PKA include CREB1, CREM, ATF1 and FOS. Hence, these transcription factors may participate in the regulation of steroidogenesis from Leydig cells.

### 5.1. CREB-Related Members

**Figure 4:**
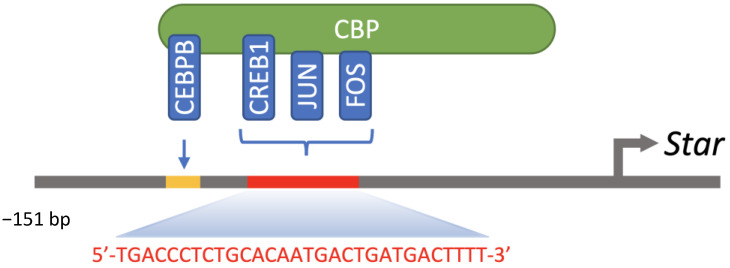
Overview of bZIP transcription factors recruited to the proximal region of the *Star* promoter. CEBPB is recruited to its regulatory element between −117 and −108 bp (5′-ATGAGGCAAT-3′), whereas CREB1, JUN and FOS form homo- or heterodimer competing for binding to the −96 to −67 bp region.

Different transcription factors, including bZIP members such as CREB1 [143] and AP-1 members [71,144], are involved in the regulation of *Star* expression in Leydig cells. The STAR protein participates in the import of cholesterol inside mitochondria, an essential step for the initiation of steroidogenesis. Three CRE half sites (−96/−67 bp) have been characterized and shown to recruit CREB1 to the cAMP-responsive region (−151/−1 bp) of the mouse *Star* promoter (Figure 4) [143]. CREB1 can also recruit the CREB regulated transcription coactivator 2 (CRTC2) to regulate *Star* expression in response to hormone and metabolic signals in Leydig cells [145,146]. PKA phosphorylates CREB1 on Ser^133^, resulting in increased recruitment to DNA and target genes expression. Activation of the cAMP/PKA pathway may not be the sole inducer of CREB1 recruitment to its DNA regulatory elements. Indeed, PMA (phorbol-12-myristate-13-acetate), an activator of the PKC pathway, increases the phosphorylation of CREB1 and induces STAR expression in MA-10 Leydig cells [12,16]. Furthermore, activations of PKA and PKC signaling pathways result in an increased association of phosphorylated CREB1 to the proximal region of the *Star* promoter [147]. However, phosphorylation of the STAR protein by PKC cannot lead to its activation, unlike PKA. Thus, the contribution of PKC to the regulation of steroidogenesis in Leydig cells requires activation of PKA for the STAR protein to be phosphorylated and functional [12]. Hormone-induced recruitment of phosphorylated CREB1 and coactivator p300 to the *Nr4a1* promoter also requires activation of the Ca^2+^/CAMKI pathway [148]. NR4A1 is an important mediator of hormone-stimulated *Star* expression [149,150]. Furthermore, silencing *Creb1* impaired NR4A1 expression and steroidogenesis in MA-10 Leydig cells [148]. NR4A1 is an orphan nuclear receptor involved in cAMP/PKA-dependent activation of steroidogenic promoters for *Star*, *HSD3B2* and *Cyp17a1* [149,151,152,153]. The initial substrate of steroidogenesis, cholesterol, is metabolised into pregnenolone by the cholesterol side chain cleavage enzyme CYP11A1 (cytochrome P450, family 11, subfamily a, polypeptide 1). Then, the enzyme hydroxy-delta-5-steroid dehydrogenase, 3 beta- and steroid delta-isomerase (HSD3B2 in human and HSD3B1 in rodents) catalyzes the conversion of pregnenolone into progesterone, whereas CYP17A1 converts progesterone into androstenedione. Testosterone is then synthesized from androstenedione by the enzyme HSD17B3.

In addition to regulating steroidogenic genes, the CREB1 transcription factor also modulates the expression of other genes encoding enzymes in response to activation of the cAMP/PKA pathway in Leydig cells. For example, the expression of acyl-CoA synthetase 4 (*Acsl4*) is up-regulated by the recruitment of CREB1 to a DNA regulatory element at −200 bp of its promoter in MA-10 Leydig cells [154]. ACSL4 catalyzes the synthesis of acyl-CoA from arachidonic acid (AA), contributing to the regulation of AA at the cellular level. AA has been widely documented as an essential regulator of LH-dependent testosterone synthesis in rat Leydig cells [155,156,157,158].

Others have reported that CREB1 plays a critical role in activating kisspeptin (*Kiss1*) expression in mouse Leydig cells leading to autocrine regulation of steroidogenesis [159]. Indeed, CREB1-deficient Leydig cells fail to induce *Kiss1* expression in response to hCG treatment.

The expression of CREB3 is increased in mouse Leydig cells from prepubertal to adult stages [160]. The siRNA-mediated knockdown of CREB3 in Leydig cells results in the up-regulations of *Star*, *Cyp11a1*, *Hsd3b1* and *Cyp17a1* genes’ expressions, leading to increased testosterone production [160]. Such CREB3-dependent regulation may involve inhibition of the nuclear receptors NR4A1 and NR5A1 [160], two nuclear receptors important for steroidogenic genes expressions. Hence, CREB3 may contribute, in part, to the decrease in testosterone production from Leydig cells according to aging by decreasing levels and activity of nuclear receptors.

The detection of CREBZF in mouse and human testis Leydig cells is debatable in vivo [26,161,162] and may be attributed to the characterization of different isoforms. However, this transcription factor is involved in hCG-stimulated testosterone production [163]. CREBZF does not bind DNA by itself as a homodimer but rather regulates transcription by heterodimerizing with other transcription factors. In response to MAPK signaling, CREBZF can interact with ATF4 to enhance its transactivation potential [164]. CREBZF can also heterodimerize with XBP1 or CREB3 to participate in the regulation of the UPR response [165]. Silencing of *Crebzf* in primary and MLTC-1 Leydig cells decreased the expression of *Cyp17a1*, *Hsd3b1* and *Hsd17b3*, resulting in the inhibition of hCG-stimulated testosterone production [163]. Interestingly, CBEBZF activates the expression of *Nr4a1* and *Nr5a1* [163].

### 5.2. AP-1 Members

In addition to PKA, other signaling pathways such as PKC and MAPK may participate in the regulation of steroidogenesis in Leydig cells by phosphorylating specific AP-1 transcription factors [15,166]. Indeed, JUN can activate the *Nr4a1* promoter following its recruitment to three AP-1-like DNA regulatory elements in MA-10 Leydig cells [6,167]. In addition, JUN has additive effects with NR4A1 to activate the *Star* promoter by being recruited to the AP-1 regulatory element at −78 bp [71]. However, the binding of JUN to this regulatory element is not altered by treatment with cAMP for up to 2 h [5]. Other nuclear receptors such as NR5A1 and NR5A2 can also cooperate with JUN to activate *Star* gene expression [71]. In addition, NR5A1 cooperates with JUN to activate the human *CYP11A1* promoter in steroidogenic cells [168]. Notably, such cooperation between JUN and nuclear receptors involves protein–protein interactions. However, others have shown that JUN rather inhibits the capacity of NR4A1 to activate the *Cyp17a1* promoter in K28 Leydig cells [169]. In this regulatory process, the JNK signaling pathway is activated by reactive oxygen species, JUN and NR4A1 physically interact, and DNA binding of NR4A1 is inhibited. JUN may also interact with other nuclear receptors, such as the glucocorticoid receptor [170], the progesterone receptor [129], the estrogen receptor [129,171], the AR [129,130], the thyroid receptor [161] and the retinoic acid/retinoid X receptors [162,172], to influence the expression of steroidogenic genes. However, the implications of these protein interactions on steroidogenesis will need further investigation.

JUN also cooperates with GATA4 to activate *Star* expression [173]. This regulation is mostly dependent on the proximal GATA DNA-regulatory element in the *Star* promoter, suggesting that these transcription factors physically interact. Although a cooperation between JUN and FOS activates the *Star* promoter, JUN homodimers are rather responsible for the cooperation with GATA4 [173]. However, FOS can still be recruited to the mouse *Star* proximal promoter region [174], suggesting that JUN/FOS heterodimers may participate in the regulation of *Star* gene expression. Interestingly, *Fosl2* overexpression rather inhibits JUN-dependent activation of the *Star* promoter [174]. Thus, the combination of AP-1 factors may finely regulate *Star* expression according to the levels of androgen synthesis by Leydig cells.

Members of the AP-1 family may regulate steroidogenesis with precision through post-translational modifications. In response to PKA activation in MA-10 Leydig cells, the levels of FOS protein are increased and phosphorylated at Thr^325^, whereas JUN is only phosphorylated at Ser^73^ after 15–30 min of dibutyryl-cAMP treatment [144]. Such post-translational modifications increase recruitment of these AP-1 members to the AP-1/CRE element located at −81 to −75 bp of the *Star* proximal promoter in mice [144]. AP-1 members JUN and FOS also promote CREB1 and CBP recruitments to the *Star* promoter. However, because AP-1 members can interact with a common DNA-regulatory element for CREB1, activation of *Star* expression by CREB1 is inhibited by FOS and JUN [144]. In addition to JUN, other AP-1 members such as FOS and JUND can also activate the *Star* promoter [71]. However, FOS rather inhibits steroidogenic gene expression in adrenal [175] and Leydig cells [144]. Precisely, AMP-activated protein kinase (AMPK)/PKC signaling pathway activation within Leydig cells represses JUN and NR4A1 but activates FOS and NR0B1 (DAX1), resulting in decreased *Star* expression and steroidogenesis [176]. Overall, JUN cooperates with several transcription factors to regulate the expression of steroidogenic genes, including specificity protein 1 (SP1) [177] and NR5A1 to regulate *Fdx1* (ferredoxin-1) [72], as well as GATA4, NR5A1 and NR4A1 to regulate *Star* [71,173], in Leydig cells. Indeed, JUN and FOS can physically interact with NR5A1, GATA4 and CEBPB as reported using the mammalian two-hybrid system [174]. Thus, AP-1 members can form major complexes contributing to the regulation of steroidogenic genes’ expressions.

Several extracellular signals participate in the activation of members of the PKC family of serine/threonine kinases. In MA-10 Leydig cells, different PKC isoforms are induced by PMA and may contribute to *Star* activation and increased progesterone synthesis [15]. In addition, PMA increases JUN and FOS protein levels and their phosphorylation [15], suggesting that the activation of *Star* expression by PMA relies on activation of AP-1 members. Indeed, PKCμ inactivation decreases the expression and phosphorylation of JUN/FOS, as well as their association with the *Star* proximal promoter region, resulting in decreased *Star* expression and progesterone synthesis in MA-10 Leydig cells [15]. Interestingly, binding of JUN/FOS to the *Star* promoter in response to PMA promotes recruitment of the CBP cofactor [15], resulting in histone acetylation and increased accessibility to the promoter for the transcriptional machinery.

Once inside the mitochondria, cholesterol is converted to pregnenolone by the rate-limiting steroidogenic side chain cleavage enzyme, CYP11A1. In mouse trophoblast and granulosa cells, the *Cyp11a1* promoter activity may be regulated by AP-1 members and GATA4 [178]. Although AP-1 regulatory elements have been characterized in the *Cyp11a1* promoter [179], their implication in transcriptional regulation in Leydig cells remains to be confirmed.

After being reduced by the flavoprotein ferredoxin reductase, ferredoxin 1 (FDX1) supports steroid biosynthesis in steroidogenic cells by transferring electrons to CYP11A1. We have reported that JUN interacts with NR5A1 and these transcription factors cooperate to activate the *Fdx1* promoter in different Leydig cell lines [72]. Such activation involve regulatory elements located within the *Fdx1* proximal promoter region. Thus, AP-1 members may contribute to the activation of the CYP11A1 enzyme by increasing *Fdx1* gene expression in Leydig cells.

The gene *Tspo* encodes the translocator protein, a cholesterol-binding protein promoting the entry of cholesterol inside mitochondria [180,181,182]. The expression of *Tspo* in Leydig cells depends on PKCε-dependent phosphorylation of JUN through the MAPK pathway [166]. Moreover, PMA, an activator of PKCε, also promotes the binding of JUN to its AP-1 DNA regulatory element in the *Tspo* promoter [183]. Such regulatory mechanism may involve the interaction between JUN and STAT3, resulting in synergistic activation of the *Tspo* promoter [166]. However, the consequences of the regulation of *Tspo* by members of the AP-1 family on steroidogenesis by Leydig cells will require further investigations.

Different growth factors produced by Sertoli cells have paracrine actions influencing Leydig cells’ functions. Among them, FGF, IGF1 and TGFα increase, whereas TGFβ decreases, androgen production from Leydig cells [184,185,186,187]. Precisely, IGF1 increases the availability of cholesterol, as well as the activities of steroidogenic enzymes, promoting the conversion of pregnenolone to testosterone [188]. Increased *Star* expression leading to progesterone synthesis has also been observed in response to stimulations of MLTC-1 cells with IGF1, FGF and TGFα [13]. Importantly, IGF1 and the cAMP/PKA pathway cooperate to activate CREB1 and JUN, leading to increased *Star* expression [189]. The expressions of the receptors for these growth factors can be regulated by AP-1 factors in Leydig cells, as shown with the upregulation of *FGFR1* by FOS in osteosarcoma cells [190]. Although several growth factors are produced by Sertoli cells, only kit ligand (KITL) and glial cell derived neurotrophic factor (GDNF) are required for male fertility. Interestingly, the transcription factors JUN and FOS may increase the expression of receptors for KITL and GDNF in Leydig cells, as indicated with the *cKIT* promoter [191].

In addition to LH, Leydig cells can also be stimulated by other hormones such as the growth hormone (GH). Indeed, GH increases *Star* gene expression and steroidogenesis by promoting cooperation between JUN and STAT5B [192]. However, it would be interesting to determine if such cooperation is also involved in the regulation of other STAT target genes such as *HSD3B2* [193] in Leydig cells.

Being part of the FOS-related family of transcription factors, the Jun dimerization protein 2 (JDP2) is highly expressed in MA-10 Leydig cells. In addition, its expression can be modulated by activation of the cAMP/PKA pathway (our unpublished observations). JDP2 binds DNA as a homodimer or as a heterodimer with ATF2 and JUN proteins, but not with FOS proteins [194]. JDP2 represses transcription either as a homo- or heterodimer [195]. However, its physiological role in regulation of steroidogenesis in testicular Leydig cells remains to be investigated further.

Overall, AP-1 members are critical regulators of the expression of genes related to steroidogenesis in Leydig cells. Precisely, the combination of expressed AP-1 factors, the post-translational modulation of their activities, and the interactions with other partners can all determine how these transcription factors influence androgen synthesis in the testis.

#### NFE2 Subfamily Members

The member of the nuclear factor, erythroid 2 (NFE2) subfamily of bZIP transcription factors NFE2L2 (NRF2) is a master regulator of phase 2 antioxidant genes and seems to play an important role in preventing the reduction in testosterone production from aging Leydig cells. Indeed, the knockout of *Nfe2l2* in mice results in a greater reduction in testosterone production by Leydig cells during aging compared to normal mice [196]. Although largely correlated, there is evidence that the redox imbalance occurring with aging may result in reduced steroid formation by Leydig cells [197]. Under oxidative stress, NFE2L2 dissociates from kelch-like ECH-associated protein 1 (KEAP1), translocates to the nucleus, and then activates NFE2L2-responsive genes involved in antioxidant activity and oxidant inactivation [198]. However, the target genes for NFE2L2 have not been clearly characterized in Leydig cells.

### 5.3. CEBP Members

CEBPB is expressed in steroidogenic cells, such as Leydig and granulosa cells [199], and plays an essential role in LH-regulated differentiation and function of Leydig cells [77]. Indeed, activations of PKA and PKC signaling pathways increase CEBPB protein levels in MA-10 Leydig cells [147]. CEBPB activates the expression of the *Star* gene, an important regulator of cholesterol import inside mitochondria [200,201]. Indeed, CEBPB can bind to the −117 to −108 bp *Star* promoter region [202,203,204,205], and can positively regulate *Star* gene basal expression [147,206]. Like other bZIP members, its transcriptional regulatory activity is enhanced by activation of the cAMP/PKA pathway [202]. In addition, CEBPB cooperates with different transcription factors, such as NR5A1 [200] and GATA4 [201], for cAMP/PKA-dependent regulation of *Star* promoter activity in MA-10 Leydig cells. It can also cooperate with nuclear factor kappa B subunit 1 (NFKB1) to regulate the expression of *Nr4a1* in Leydig cells [207].

The prostaglandin endoperoxide synthase 2 (PTGS2, COX2) enzyme, involved in the synthesis of PGF_2α_ and PGE_2_, is highly expressed by Leydig cells from infertile patients [208] as well as during aging [209]. In MA-10 Leydig cells, CEBPB is recruited to a regulatory element in the proximal region of the *Ptgs2* promoter resulting in activation of gene expression in response to cAMP [135]. DDIT3, an inhibitor of CEBP family members by heterodimer formation [136], is also expressed in MA-10 cells [135]. In contrast to CEBPB, DDIT3 expression is rather reduced in response to hCG or cAMP [135]. Thus, induction of *Ptgs2* expression correlates with increased *Cebpb* and reduced *Ddit3* expressions in response to activation of the LH/cAMP/PKA pathway. Such activation of *Ptgs2* by CEBPB may contribute to decreased steroid production by aging Leydig cells.

### 5.4. Transmembrane bZIP Transcription Factors

In addition to the bZIP domain, several transcription factors also contain a transmembrane domain and participate in ER unfolded protein stress transduction. These bZIP transcription factors include ATF6 and the CREB3-like subfamily [210]. Since a major part of steroidogenesis is taking place in the ER, the UPR may have an indirect influence on gene regulation through activation of ER transmembrane bZIP transcription factors, leading to their migration in the nucleus. However, such gene regulation will require further investigation. Prolonged exposure to a high dose of hCG induces ER stress and downregulation of steroidogenic enzyme gene expression in Leydig cells [89]. Such effect may be attributed to the triggering of UPR and nuclear translocation of ATF6 in Leydig cells [89]. Interestingly, other bZIP transcription factors, such as CREB3L4, are involved in ER stress response and are crucial for proper spermatogenesis and spermiogenesis [211,212].

## 6. bZIP Transcription Factors and INSL3

Apart from producing steroid hormones, testicular Leydig cells are also the main source of the peptide hormone insulin-like 3 (INSL3). This hormone regulates the transabdominal phase of the descent of testes in the scrotum during fetal development as well as germ cell survival [213]. However, INSL3 is not required for spermatogenesis and germ cells’ survival in adult mice [214]. The production of INSL3 is dependent on the differentiation state of Leydig cells and is correlated with the circulating levels of LH [215,216,217,218]. Intrestingly, differentiated Leydig cells produce and secrete INSL3 constitutively. The *Insl3* gene promoter contains several DNA regulatory elements for ATF3, CREB1 and MAF, suggesting a possible regulation by bZIP members. However, the regulation of *Insl3* expression by these transcription factors may be indirect and involve activation of *Nr4a1* expression, as reported for JUN [157,174,219].

## 7. bZIP Transcription Factors and Leydig Cell Communication

The regulation of steroidogenesis in Leydig cells depends not only on the activation of receptors associated with various signaling pathways but may also rely on the exchange of signaling molecules via gap junctions between adjacent cells. Gap junctions are formed by connexin proteins that assemble into homomeric or heteromeric connexons to create a channel for exchange of cytosolic molecules between neighboring cells. Different types of connexins are encoded by a class of genes including five groups (*Gja*, *Gjb*, *Gjc*, *Gjd* and *Gje*), each composed of several members and isoforms. Leydig cells express mainly *Gja1* (CX43), and to a lesser extent *Gja4* (CX37), *Gja6* (CX33), *Gjc1* (CX45), *Gjc2* (CX47), *Gjd2* (CX36), and *Gjd3* (CX31.9/30.2) [220,221,222,223]. Only the inactivation of GJA1 has been characterized for its effects on steroidogenesis and does not appear to be critical for Leydig cell development, differentiation and function [224,225]. This may be attributed to the functional redundancy between different types of connexins in Leydig cells [222]. However, GJA1 inactivation in Sertoli cells results in Leydig cell hyperplasia [226,227], suggesting that this connexin is involved in paracrine communication between these cell types. Interestingly, this phenotype is also associated with a decrease in *Gja1* and *Gjc1* expressions in Leydig cells, without any effect on steroidogenesis [228]. As reported in prostaglandin E2 treated cells [219], GJA1-dependent potentiation of cAMP/PKA signaling in Leydig cells is not associated with an increase in cAMP levels, but rather with an increased exchange of this second messenger between interconnected cells.

### 7.1. CREB Members

The expression of *Gja1* has been reported to be regulated by CREB1 in other cell types. Indeed, β-adrenoceptor stimulation upregulates cardiac *Gja1* expression via PKA and MAPK signaling pathways, possibly through activation of AP1 and CREB1 factors [229]. In addition, a complex composed of NFKB, CREB1 and CBP is formed following oxytocin stimulation and participates in the activation of *Gja1* expression in mouse embryonic stem cells [230]. It has been reported that hCG and 8-bromo-cAMP treatments, known to increase *Cyp11a1* and *Star* expression, inhibit *Gja1* expression in rat Leydig cells [231]. However, others have shown that these treatments rather increase GJA1 levels in cultured Leydig cells [232], suggesting that junctional coupling between Leydig cells is hormonally regulated. Indeed, *Gja1* expression and cell-to-cell communication are modulated by long-term (36 h) stimulation with LH or hCG [232]. Furthermore, GJA1 expression in postnatal Leydig cells has been correlated with testosterone production at different stages of postnatal development [233]. Indeed, *Gja1* expression peaks at P40 and remains elevated throughout adulthood in rat Leydig cells [231]. Thus, gap junctions may enhance testosterone production by facilitating the exchange of cAMP between neighboring cells to increase steroidogenic gene expression. However, the implication of CREB1 in the regulation of *Gja1* expression in Leydig cells remains to be confirmed.

### 7.2. AP-1 Members

**Figure 5:**
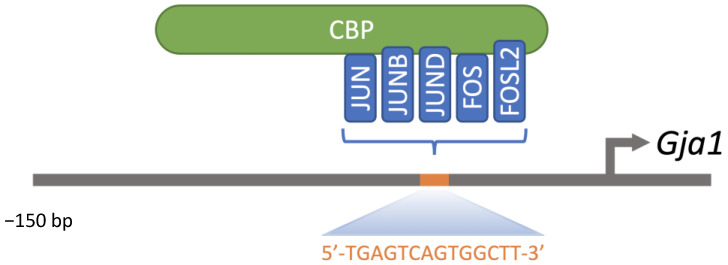
Overview of bZIP transcription factors recruited to the proximal region of the *Gja1* promoter. JUN, JUNB, JUND, FOS and FOSL2 can participate in the formation of homo- or heterodimers to be recruited to the −45 bp AP-1 DNA regulatory element.

AP-1 members JUN and FOS are major regulators of *Gja1* expression in mouse TM3 Leydig cell line [234]. Such synergy involves the recruitment of FOS to an AP-1 DNA-regulatory element located in the proximal region of the *Gja1* promoter (Figure 5). This element is highly conserved among species, suggesting that the ability of AP-1 members to regulate *Gja1* expression has been conserved throughout evolution. In addition, AP-1 members may be functionally redundant as JUN, JUNB and FOSL2 also efficiently increased *Gja1* promoter activity in TM3 Leydig cells [234]. With significant endogenous levels in Leydig cells [223,235], FOS or FOSL2 can interact with JUN, JUNB or JUND to regulate *Gja1* expression. Thus, AP-1 dimers binding to the *Gja1* promoter may consist of FOS/JUN, FOS/JUNB, FOS/JUND, FOSL2/JUN, FOSL2/JUNB or FOSL2/JUND in mouse Leydig cells. AP-1 members are therefore important for optimal *Gja1* expression in testicular Leydig cells. In addition to variations in the abundance of FOS and JUN proteins in Leydig cells, activation of the *Gja1* promoter may also involve their post-translational modifications by protein kinases and their interactions with other partners or coactivators [121].

#### 7.2.1. NFE2 Subfamily Members

In response to treatments with the polyphenolic compound luteolin, the transcription factor NFE2L2 translocates into the nucleus of TM4 Sertoli cells and is associated with an increased expression of *Gja1* [236]. However, even though the *Gja1* promoter contains several NFE2L2 DNA regulatory elements in its proximal region, the recruitment of NFE2L2 to this region remains to be confirmed. In addition, such regulation of *Gja1* expression by NFE2L2 in Leydig cells should be characterized further.

#### 7.2.2. ATF3-like Subfamily Members

As reported previously, JDP2 is highly expressed in MA-10 Leydig cells and is inducible following activation of the cAMP/PKA pathway (our unpublished observations). Interestingly, JDP2 overexpression in myocardial cells results in loss of *Gja1* expression [237], suggesting that such regulatory mechanism may be present in Leydig cells. Moreover, several JDP2 DNA regulatory elements can be found in the proximal region of the *Gja1* promoter.

### 7.3. MAF-Related Members

In chicken embryos, overexpression of Maf and Mafa induced ectopic expression of *Gja1* [238]. In human testis, MAF is highly expressed in Leydig cells and cells from the seminiferous tubules [26]. In addition, the *Gja1* promoter contains several MAF regulatory elements in its proximal region. However, the molecular implication of MAF transcription factors in the regulation of *Gja1* expression in Leydig cells remains to be investigated.

### 7.4. CEBP Members

In breast cancer cells, CEBPA regulates *GJA1* expression by binding to the DNA regulatory element 5′-AATTGTC-3′ at −456 bp of the promoter region [239]. However, CEBPA expression in Leydig cells appears to be very low [26]. Thus, its role in the regulation of *Gja1* expression may be replaced by CEBPB but remains to be confirmed.

## 8. Conclusions

Significant progress has been made over the past 40 years in our knowledge of the signaling pathways and the underlying processes that control the activity of bZIP transcription factors in different cell types. As highlighted in this review, CREB, CEBP and AP-1 members of the bZIP family play important roles in regulating gene expression critical for adequate Leydig cell function in the testis. Their specificity of action in Leydig cells relies on bZIP-dependent homo- and heterodimer formation, as well as interactions with other types of transcription factors or cofactors. Hence, major questions remain regarding the mechanisms of action of bZIP members in Leydig cells and include: What are the contributions of signaling pathways in the activation of bZIP members and in the formation of dimers leading to the regulation of Leydig cell-specific target genes? What is the importance of redundancy between bZIP members in the formation of heterodimers? Can heterodimers between bZIP members vary depending on the target gene, cellular context, or signaling pathways activated? How is the binding of bZIP factors to their DNA regulatory elements coordinated with the recruitment of other transcription factors nearby, and what are the consequences of these recruitments to DNA on transcriptional regulation? Answers to these questions are essential to a better understanding of how transcription factors of the bZIP class influence gene expression in Leydig cells.

## Figures and Tables

**Table 1:** Classification of transcription factors members of the class of bZIP transcription factors according to the TFClass nomenclature [1].

Family	Subfamily	Transcription Factors
JUN-related	JUN	JUN, JUNB, JUND
	NFE2	NFE2, NFE2L1, NFE2L2, NFE2L3, BACH1, BACH2
	ATF2	ATF2, ATF7, CREB5
FOS-related	FOS	FOS, FOSB, FOSL1, FOSL2
	ATF3-like	ATF3, JDP2
MAF-related	Large MAF	MAF, MAFA, MAFB, NRL
	Small MAF	MAFF, MAFG, MAFK
B-ATF-related		BATF, BATF2, BATF3
XBP1-related		XBP1
ATF4-related		ATF4, ATF5
CREB-related	CREB-like	CREB1, ATF1, CREM
	CREB3-like	CREB3, CREB3L1, CREB3L2, CREB3L3, CREB3L4
	ATF6	ATF6, ATF6B
	CREBZF-like	CREBZF
	CREBL2-like	CREBL2
CEBP-related	CEBP	CEBPA, CEBPB, CEBPG, CEBPD, CEBPE, DDIT3
	PAR	DBP, HLF, NFIL3, TEF

## Data Availability

Not applicable.
